# Glucagon‐like peptide‐1 receptor agonists (GLP‐1 RAs) for the management of nonalcoholic fatty liver disease (NAFLD): A systematic review

**DOI:** 10.1002/edm2.163

**Published:** 2020-06-11

**Authors:** Xiaodan Lv, Yongqiang Dong, Lingling Hu, Feiyu Lu, Changyu Zhou, Shaoyou Qin

**Affiliations:** ^1^ Department of Endocrinology China‐Japan Union Hospital of Jilin University Changchun China; ^2^ Department of Thyroid Surgery The First Affiliated Hospital of Zhengzhou University Zhengzhou China; ^3^ Department of Endocrinology Ningbo Medical Center Lihuili Eastern Hospital Zhejiang China; ^4^ Department of Paediatrics The First Hospital of Jilin University Changchun China; ^5^ Department of Gastroenterology and Hepatology China‐Japan Union Hospital of Jilin University Changchun China

**Keywords:** glucagon‐like peptide‐1 receptor agonists, nonalcoholic fatty liver disease, type 2 diabetes

## Abstract

There are no licensed drugs for nonalcoholic fatty liver disease (NAFLD), and there is a lack of consensus on the best outcome measures for controlled trials. This systematic review aimed to evaluate the efficacy of GLP‐1 RAs in the management of NAFLD, the degree of heterogeneity in trial design and the robustness of conclusions drawn from these clinical trials. We searched publication databases and clinical trial registries through 2 November 2019 for clinical trials with NAFLD. We evaluated improvements in histological findings, noninvasive markers of hepatic steatosis, inflammation, and fibrosis, insulin resistance and anthropometric measures. Our final analysis included 24 clinical trials, comprising 6313 participants with a mean duration of 37 weeks. Four clinical trials, including RCT (n = 1), single‐arm studies (n = 2) and case series studies (n = 1), used biopsy‐confirmed liver histological change as their end‐points. The remaining studies (n = 20) used surrogate end‐points. GLP‐1 RAs were effective for the improvement in hepatic inflammation, hepatic steatosis and fibrosis. More importantly, GLP‐1 RAs showed promise in improving the histological features of NASH. In addition, 8 ongoing trials were identified. In this systematic review of published and ongoing clinical trials of the efficacy of GLP‐1RAs for NAFLD, we found that GLP‐1 RAs are effective for hepatic steatosis and inflammation, with the potential to reverse fibrosis. Further prospective studies of sufficient duration using histological end‐points are needed to fully assess the efficacy of GLP‐1 RAs in the management of NAFLD.

## Graphical abstract

This systematic review of published and ongoing clinical trials found that GLP‐1 RAs are effective for hepatic steatosis and inflammation, with the potential to reverse fibrosis. Further prospective studies of sufficient duration using histological end‐points are needed to fully assess the efficacy of GLP‐1 RAs in the management of NAFLD.

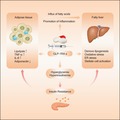

## 1 INTRODUCTION

**FIGURE 1:**
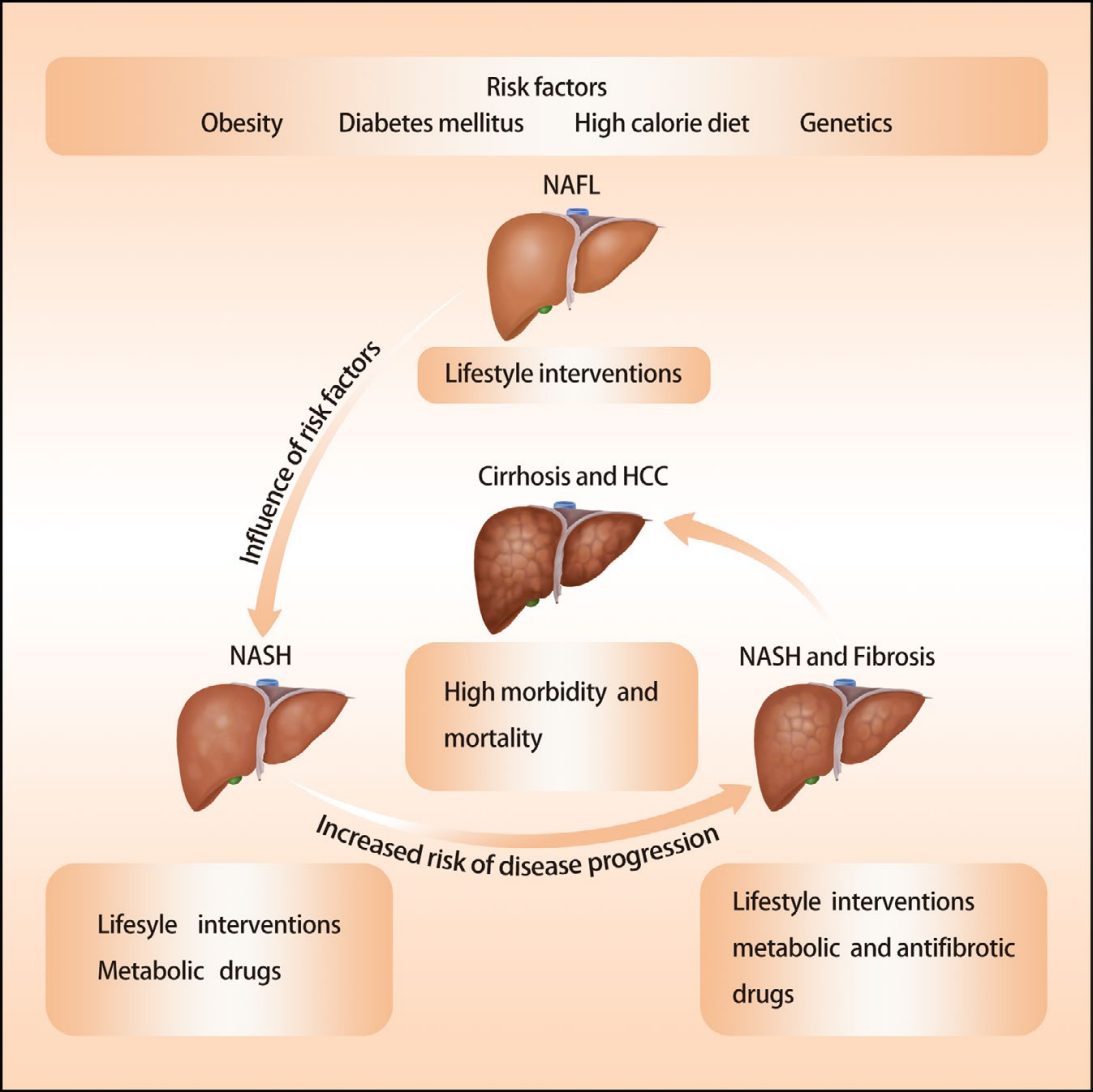
Natural history, risk factors and treatment approaches of NAFLD

Nonalcoholic fatty liver disease (NAFLD) is the most common chronic liver disease with a global prevalence of 25.2%,^1^ and a higher prevalence of 55.5% in patients with type 2 diabetes mellitus (T2DM).^2^ NAFLD is divided into two histological subtypes of (a) nonalcoholic fatty liver (NAFL), characterized by isolated hepatic steatosis, often with mild nonspecific inflammation, and (b) nonalcoholic steatohepatitis (NASH), characterized by the presence of hepatic steatosis and hepatocellular injury with or without fibrosis. NASH is considered to be the more severe form of NAFLD. Approximately 20% of individuals with NASH can progress to cirrhosis, liver failure and hepatocellular carcinoma, while less than 4% of individuals with NAFL progress to cirrhosis.^3^, ^4^, ^5^ Patients with T2DM are particularly susceptible to NASH, with a higher risk of progressing into cirrhosis and hepatocellular carcinoma.^6^, ^7^, ^8^, ^9^, ^10^ Moreover, the coexistence of NAFLD and T2DM is not only associated with a worse liver outcome but also related to increased risk of extrahepatic diseases, such as cardiovascular disease and chronic kidney disease.^11^, ^12^ Therefore, altering the natural course of NAFLD, particularly in T2DM patients, is vital for reducing the health and economic burden of NAFLD and NAFLD‐related extrahepatic diseases (Figure 1).

Lifestyle intervention, the first line of treatment for T2DM and obesity, has proven to be effective in the management of NAFLD. Reduction of 5%‐10% in body weight with life modification over 24‐48 weeks leads to a significant improvement in hepatic steatosis, necroinflammation and even fibrosis.^13^, ^14^, ^15^, ^16^, ^17^ However, lifestyle intervention alone rarely achieves a complete resolution of NASH and it is challenging to maintain long‐term weight loss. Therefore, many pharmacological interventions have been investigated to limit the development and progression of NAFLD, although there are no currently licensed drugs for the treatment of NAFLD.^17^, ^18^

Given the close association between NAFLD and T2DM, the effect of antidiabetic medicine for the treatment of NAFLD has attracted substantial scientific attention.^18^, ^19^, ^20^, ^21^, ^22^, ^23^ Many clinical trials have suggested the emerging role of glucagon‐like peptide‐1 receptor agonists (GLP‐1 RAs) in the management of NAFLD. However, one of the biggest challenges in designing and implementing controlled trials in NAFLD is the lack of consensus on appropriate end‐points for assessing the benefit of GLP‐1 RAs for NAFLD.^24^, ^25^ Although end‐points for NAFLD in clinical trials have evolved during the past decades, liver biopsy is still the gold standard for diagnosis and assessment of NAFLD. However, the invasive nature of liver biopsy and reluctance from patients limits its use in clinical trials and thus constitutes a major barrier for drug development in NAFLD. As a result, several noninvasive serum markers or imaging modalities for diagnosis or assessing response to treatment for NAFLD have been developed, and they have been increasingly used for defining end‐points in clinical trials.^26^, ^27^, ^28^, ^29^, ^30^, ^31^

Our systematic review aimed to evaluate the efficacy of currently available GLP‐1 RAs (Table 1) in the management of NAFLD, the degree of heterogeneity in trial design and the robustness of conclusions drawn from these clinical trials.

**TABLE 1:** Currently approved GLP‐1 RAs

	Exenatide (LAR)	Liraglutide	Exenatide (ER)	Albiglutide	Dulaglutide	Lixisenatide	Semaglutide (Injection)	Semaglutide (OA)
Brand name	Byetta	Victoza	Bydureon	Tanzeum	Trulicity	Adlyxin	Ozempic	Rybelsus
Company	AstraZeneca	Novo Nordisk	AstraZeneca	GlaxoSmithKline	Eli Lilly and company	Sanofi	Novo Nordisk	Novo Nordisk
FDA approval date	2005	2010	2012	2014	2014	2016	2017	2019
Dose and administration	5‐10 ug SC, twice daily, prior to meals	0.6‐1.8 mg SC, once daily independent of meals	2 mg SC, once weekly, independent of meals	30‐50 mg SC, once weekly independent of meals	0.75‐1.5 mg SC, once weekly independent of meals	10‐20ug SC, once daily, 1 h before the first meal of the day	0.25‐1 mg SC, once weekly, independent of meals	3‐14 mg PO once daily, at least 30 min before the first food
Elimination half‐life	2.4 h	13 h	2.4 h once release	5 d	4.5 d (0.75 mg) 4.7 d (1.5 mg)	3 h	1 wk	1 wk

Abbreviations: LAR, immediate release; ER, extended release; SC, subcutaneous injection; OA, oral administration; PO, per os.

## 2 METHODS

### 2.1 Data sources and extraction

This systematic review was performed in accordance with the Preferred Reporting Items for Systematic Reviews and Meta‐Analyses (PRISMA) guidelines.^32^ We conducted a systematic literature search of PubMed, Scopus, Web of Science, ClinicaTrials.gov, Cochrane CENTRAL Register of Controlled Trials and World Health Organization International Clinical Trials Registry. The finalized searches were performed on 2 November 2019. The search terms included glucagon‐like peptide‐1 receptor agonists, dulaglutide, exenatide, liraglutide, lixisenatide, semaglutide, albiglutide, NAFLD, NASH and NAFL. The complete search strategy independently verified by individuals (XDL, YQD) was included in Supplemental Material 1. Additionally, we reviewed references from included original papers to identify further eligible studies. Data extraction was also independently performed by 2 authors (XDL and YQD). Differences were resolved by discussion with SYQ.

### 2.2 Selection of published studies

The inclusion criteria were published clinical trials investigating the effect of GLP‐1 RAs on NAFLD. The diagnosis of NAFLD was based on the detection of steatosis either by imaging or by histology, and appropriate exclusion of other liver diseases.^33^ The exclusion criteria were studies not written in English and those with secondary causes of hepatic steatosis. Reviews and editorials were excluded. There were no restrictions on sex, age, ethnicity and numbers of participants.

### 2.3 Selection of ongoing registered clinical trials

ClinicalTrials.gov was searched to identify ongoing registered clinical trials. The inclusion and exclusion criteria were the same as those for the selection of published studies.

### 2.4 Outcome measures

The primary outcome assessed in clinical trials included histological improvement in NAFLD, defined as the resolution of steatohepatitis without worsening of fibrosis. Secondary histological outcomes included steatosis, hepatocyte ballooning, (lobular or portal) inflammation and the combined NAFLD activity score. Other secondary outcome measures included changes in serum hepatic enzymes level, noninvasive hepatic biomarkers (APRI score, FIB‐4 score and FLI), insulin resistance (fasting homeostasis model of assessment of insulin resistance [HOMA‐IR]) and anthropometric measures.

### 2.5 Quality assessment

The quality of randomized control trials (RCTs) was assessed based on a modified version of the Cochrane Collaboration Risk of Bias Tool.^34^

## 3 RESULTS

### 3.1 Study characteristics and quality assessment

Database searches identified 1,933 published articles. 472 were excluded after duplicates removed, 1352 were excluded at the screening stage, and 85 were excluded on the full‐text review (Supplementary Material 2). A total of 24 clinical trials, including randomized controlled studies (RCTs, n = 14), parallel‐group uncontrolled studies (n = 2), observational studies (n = 1), retrospective studies (n = 1), single‐arm studies (n = 3), case series studies (n = 1) and post hoc analysis (n = 2), were finally included in this systematic review (Table 2). A total of 6313 participants were studied, with a mean duration of 37 (12‐144) weeks.

**TABLE 2:** Characteristics and findings of clinical trials of GLP‐1 RA therapy for NAFLD

Author	Study design	Number & Dose of participants per intervention	Duration (week)	Response			Tolerability	Comments
				Liver enzymes	Liver fat by imaging	Histology		
John et al,^62^ 2007; USA	RCT + Open‐label extension	Phase 1 (RCT): 1446 Exe + PLA Phase 2 (open label) 974 open label	96	Individuals with elevated ALT at baseline had a significant mean reduction in ALT.	NA	NA	Phase 1:22.2% dropout Phase 2:45.7% dropout	The high dropout rate might affect the outcome.
Klonoff et al,^94^ 2008; USA	RCT + open‐label extension	217 patients completed 3 years of exenatide exposure	>144	Individuals with elevated ALT had a significant reduction in ALT, and 41% achieved normal ALT.	NA	NA	NA	Exenatide significantly Improved a number of cardiovascular risk factors.
Jendle et al,^58^ 2009; USA	RCT	20 PLA 37 Glim 4 mg 35 Lira 0.6 mg 31 Lira 1.2 mg 37 Lira 1.8 mg (Met as baseline treatment)	26	No significant improvement with Lira than PLA.	Fat percentage with liraglutide 1.2 and 1.8 mg was significantly reduced vs. glimepiride.	NA	3.7% dropout	The liver‐to‐spleen attenuation ratio was used as an index of liver fat.
Kenny et al,^87^ 2010; USA	Case series	8 Exe (5‐10 μg, bid)	28	Mean ALT was significantly improved from 69 to 45 IU/L (p＝0.036)	NA	No significant improvement.	No dropout	Liver histology was improved in 3 of 8 patients.
Sathyanarayana et al,^74^ 2011; USA	RCT	10 Pio 45 mg 11 Pio 45 mg + Exe (10 μg, bid) (Diet as baseline treatment in both groups)	50	Both groups significantly reduced the level of ALT and AST, with a significantly greater reduction in ALT with Pio 45 mg + Exe treatment.	Reduced LFC (^1^H‐MRS) with Pio therapy (11.0 ± 3.1 to 6.5 ± 1.9%, *P* < .05), and significant greater reduction with ex + pio therapy (12.1 ± 1.7 to 4.7 ± 1.3%, *P* < .001)	NA	No dropout	Both groups significantly reduced the level of TG (*P* < .05 in Exe and *P* < .01 in Pio), with a greater reduction in the Exe group (*P* < .01).
Ohki et al,^81^ 2012; Japan	Retrospective studies	26 Lira 0.9 mg 20 Pio 15 mg 36 Sita 100 mg	48	Lira decreased AST (50 to 35 IU/L) and ALT (65 to 48 IU/L, *P* < .01). Sita decreased ALT (75 to 61 IU/L, *P* = .03).	NA	NA	No dropout	Lira significantly reduced APRI index (0.73 to 0.49, *P* < .01)
Cuthbertson et al,^68^ 2012; Italy	Observational studies	19 Exe 10 ug bid 6 Lira 1.2 mg qd	25	Mean ALT was improved from 40 to 31 IU/L (*P* < .05) and GGT improved from 69 to 43 IU/L (*P* < .01)	Mean LFC (^1^H‐MRS) was reduced from 28% to 21% (*P* < .001)	NA	19.4% dropout	The relative reduction in LFC correlated with HbA_1_c (*P* < .05).
Suzuki et al,^82^ 2013; Japan	Single‐arm study	59 Lira 0.9 mg (8 of the 59 treated with Pio as a pretreatment)	25	NA	The liver/kidney (CT) ratio was improved from 1.64 ± 0.44 to 1.78 ± 0.42.	NA	23.7% dropout	Lira alone significantly decreased the subcutaneous but not visceral fat areas.
Fan H et al,^60^ 2013; China	RCT	49 Exe 10 μg bid 68 Met 0.5 g bid	12	Both groups showed significant reduced ALT. Exe was associated with a significantly greater reduction than Met in ALT (27.32 ± 15.96 vs 12.85 ± 11.38 IU/L, *P* = .002) and AST (7.89 ± 7.87 vs 5.11 ± 6.98 IU/L, *P* = .048).	The proportion of patients with improvement in fatty liver (US) was comparable between the two groups.	NA	18.7% dropout	Exe is superior to Met in reducing body weight.
Shao, et al,^75^ 2014; China	RCT	30 Exe 10 μg bid + insulin glargine 30 Insulin aspart + insulin glargine	12	ALT, AST and γ‐GGT were significantly decreased in two groups, and Exe was associated with a lower level of hepatic enzymes than Ins (*P* < .001).	The reversal rate of fatty liver (US) in the Exe group was significantly higher than that in the Ins group (93.3% Exe vs. 66.7% Ins, *P* < .001)	NA	No dropout	FBG, PBG, HbA_1c_, TC, TG and TBIL were significantly decreased in both groups.
BlaslovK et al,^65^ 2014; Croatia	Open‐label parallel‐group uncontrolled study	87 Exe 10μg bid + Met or/and SU 38 OHA (Met or/and SU)	25	ALT was improved in both Exe and OHA groups (−4 vs. 0, *P* = .04).	NA	NA	No dropout	ΔFLI improved in Exe and OHA ( −25.95 ± 23.15 vs‐11.01 ± 25.48, *P* = .003)
Yan Bi et al,^71^ 2014; China	RCT	11 Exe 10 ug bid 11 Pio 45mg 11 Ins	26	NA	LFC (^1^H‐MRS) was significantly reduced in Exe, Pio and Ins groups (−68 ± 6%, *P* = .004 vs. −58 ± 9%, *P* = .012 vs. −49 ± 9%, *P* = .039). However, no significant difference in LFC between three groups (*P* = .454).	NA	No dropout	ΔLFC is related to ΔHbA1c and Δweight. Early metabolic control plays a vital role in slowing progression of fatty liver in T2DM.
Eguchi et al,^83^ 2015; Japan	Single‐arm	10 Lira 0.9 mg qd	96	ALT was improved from 59.7 ± 64.6 to 34.1 ± 21.7 IU/L, (*P* < .01), and AST improved from 46.9 ± 42.1 to 29.5 ± 10.4 IU/L (*P* < .01)	Liver/spleen ratio (CT) improved from 0.92 ± 0.30 to 1.04 ± 0.24, *P* < .01	Histological inflammation improved in 7 of the 10, liver fibrosis improved in 6 of the 10, and NAFLD activity score improved in 8 of the 10.	14.8% dropout	Lira has a good safety profile.
Tang et al,^59^ 2015; Canada	RCT	18 Lira 1.8 mg qd 17 Insulin glargine	12	No improvements in both groups.	Ins was associated with a significant decrease in liver mean MRI‐PDFF (13.8% to 10.6%, *P* = .005). Lira did not change MRS‐PDFF (*P* = .80).	NA	4 of Lira discontinued due to adverse effects.	Weight was improved (−2.8 ± 6.5 in Lira vs. 0 in Ins, *P* = .03)
Armstrong et al,^64^ 2016; UK	RCT	26 Lira 1.8 mg qd 26 PLA	48	Serum γ‐GGT level significantly differed between liraglutide and placebo groups. No significant difference was detected in the change in serum ALT and AST.	NA	Lira was associated with significantly increased odds of resolution of definite NASH and progression of fibrosis than placebo group.	13.46% dropout	Most adverse events were mild to moderate in severity, transient and similar between groups.
Smits et al,^84^ 2016; Netherlands	RCT	17 Lira 1.8mg qd 18 Sita 100 mg 17 PLA	12	There is no significant improvement in ALT, AST and GGT across three groups.	There is no significant improvement in hepatic steatosis (^1^H‐MRS) across three groups.	NA	1.9% dropout	Neither liraglutide nor sitagliptin affected NFS, FIB‐4 or APRI compared with the placebo.
Dutour et al,^66^ 2016; France	RCT	22 Exe 10 μg bid 22 PLA	26	NA	Exe induced a significant reduction in LFC (^1^H‐MRS) in the Exe group than in the PLA group (−23.8 ± 9.5% vs + 12.5 ± 9.6%, *P* = .007)	NA	13.6% dropout	Longer exposure time to exenatide might be needed to reveal significant improvement in myocardial triglyceride content.
Yuya Seko et al,^54^ 2017; Japan	Single‐arm study	15 Dula 0.75mg once weekly	12	ALT was improved from 52.1 ± 7.2 to 41.1 ± 6.1 IU/L, *P* = .003), and AST was improved from 50.4 ± 6.0 to 41.9 ± 5.0, *P* = .030)	Liver steatosis (CAP) was not improved.	Only one case had a liver biopsy. The total NAFLD activity score was improved from 6 to 2.	13.3% dropout	Liver stiffness was significantly improved from 9.3 ± 1.9 to 6.9 ± 1.2 kPa (*P* = .043).
Khoo et al,^70^ 2017; Singapore	RCT	12 Lira 3 mg qd 12 De	26	Both Lira and De groups had significant (*P* < .01) and similar reductions in ALT (−42 ± 46 vs. −34 ± 27 IU/L, *P* = .52) and AST.	Both Lira and De groups had significant (*P* < .01) and similar reductions in LFC (MRI‐PDFF) (−8.9 ± 13.4 vs −7.2%±7.1%, *P* = .70).	NA	No dropout	Both groups had significant reductions in liver stiffness (*P* = .003). No significant difference existed between groups.
Petit et al,^69^ 2017; France	Non‐RCT	68 Lira 1.2 mg qd 16 Ins	26	Lira was associated with a significant reduction in mean ALT (45.9 ± 23.8 to 39.5 ± 16.6 IU/L, *P* = .021) and in mean GGT (70.8 ± 91.5 to 46.0 ± 30.7 IU/L, *P* = .017)	Lira reduced LFC (^1^H‐MRS) from 17.3 ± 10.9 to 11.9 ± 9.3 (*P* < .01), corresponding to a mean 31% relative decrease in LFC.	NA	15.0% dropout	The effect of Lira in reducing LFC was mainly driven by bodyweight reduction.
Feng et al,^57^ 2017; China	RCT	29 Gli 120 mg qd 29 Lira 1.8 mg qd 29 Met 1000 mg, bid	24	ALT significantly improved in all arms, whereas AST only improved in Lira and Met groups.	LFC was significantly reduced in all groups, from 36.70%±3.65% to 13.11 ± 1.84% in the Lira group, from 32.99 ± 3.51% to 19.59 ± 2.12% in the Gli group, and from 35.13 ± 2.34% to 18.44 ± 2.20% in the Met group. Lira was associated with a more significant reduction in LFC than Gli.	NA	6.4% dropout	LFC was quantified by the ultrasonography hepatic/renal ratio. Changes in LFC were positively linked to reductions in hepatic enzymes and triglyceride levels.
Tian. F et al,^61^ 2018; China	RCT	52 Lira 1.2 mg qd 75 Met 1.0‐1.5 g, bid	12	ALT significantly improved in both groups. Lira is superior to Met for decreasing the level of ALT.	Lira and Met were linked to a markedly lower prevalence of NAFLD (US) (78.8%, 89.3%, respectively), but there is not significant difference between groups.	NA	1.50% dropout	Nine patients in the Lira group experienced slight‐to‐moderate gastrointestinal disturbances.
K.Cusi et al,^18^ 2018; Multicentre	Post hoc analysis	971 Dula 1.5 mg qw 528 PLA	24	Dula significantly reduced ALT, AST and GGT levels vs placebo [least squares mean treatment differences: –1.7 IU/L(–2.8, –0.6), *P* = .003; –1.1 IU/l (–2.1, –0.1), *P* = .037; –6.6 IU/L (CI –12.4, –0.8), *P* = .025, respectively]	NA	NA	6.7% to 29.9% dropout	In population with ALT ≥ ULN, more pronounced reductions from baseline in ALT were observed with dulaglutide vs placebo (–8.8 IU/L vs –6.7 IU/L).
Newsome et al,^55^ 2018; Multicentre	Post hoc analysis(Data from two RCTs)	718 Sema 0.05‐0.4 mg/day 103 Lira 3.0 mg 136 PLA	52	Both trials have shown dose‐dependent decreases in ALT.	NA	NA	19.85% dropout	The maximal declines in ALT occurring by approximately week 28.

Abbreviations: ALT, alanine aminotransferase; AST, aspartate aminotransferase; CT, computed tomography; De, die exercise; Dula, dulaglutide; Exe, exenatide; FBG, fasting blood glucose; FIB‐4, fibrosis 4 score; FLI, fatty liver index; GGT, gamma‐glutamyl transpeptidase; Gli, gliclazide; Glim, glimepiride; HAb1c, glycosylated haemoglobin; Ins, insulin; LFC, liver fat content; Lira, liraglutide; Met, metformin; MRS, magnetic resonance spectroscopy; NA, not assessed; OHA, oral hypoglycaemic agents; PBG, postprandial blood glucose; PDFF, proton density fat content; Pio, pioglitazone; PLA, placebo; Sema, semaglutide; Sita, sitagliptin; SU, sulphonylureas; TBIL, total bilirubin; TC, total cholesterol; TG, triglyceride; ULN, upper limit of normal.

For 14 RCTs, a total of 3449 participants were studied, with a mean intervention duration of 38 (12‐144) weeks. RCTs were evaluated based on the Cochrane Collaboration Risk of Bias Tool (Supplementary Material 3). The quality assessment found that random sequence generation was adequate in 100% (14 of 14), whereas allocation concealment was adequate in 14% (2 of 14).

### 3.2 Study design and selection of end‐points

Four of 24 studies, including RCT (n = 1), single‐arm studies (n = 2) and case series studies (n = 1), used biopsy‐confirmed liver histological change as their end‐point. The remaining studies (n = 20) used surrogate end‐points, including change in hepatic enzymes (n = 21), noninvasive assessment of hepatic steatosis (n = 8) and liver fibrosis (n = 9).

### 3.3 Study interventions

**FIGURE 2:**
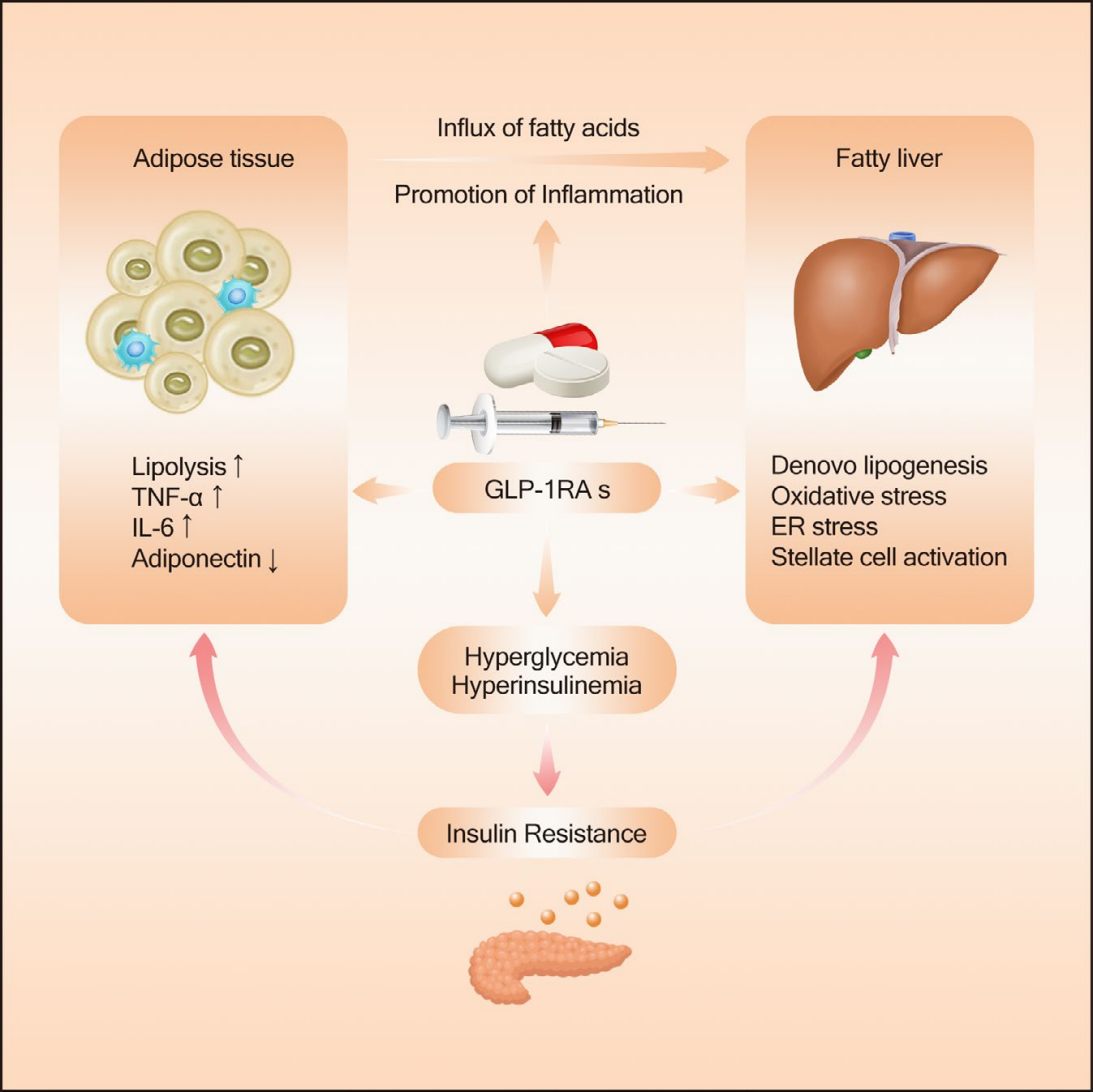
The mechanism of action of GLP‐1 RAs for the treatment of NAFLD

GLP‐1 RAs are a class of antidiabetic agents that mimic the actions of the endogenous glucagon‐like peptide. GLP‐1 RAs have been shown to reduce insulin resistance, which is strongly associated with the development and progression of NAFLD ^35^, ^36^, ^37^, ^38^, ^39^, ^40^, ^41^, ^42^, ^43^, ^44^, ^45^, ^46^, ^47^, ^48^, ^49^ (Figure 2). Many studies have demonstrated the efficacy of GLP‐1 RAs in the management of T2DM and obesity, and the potential of GLP‐1 RAs for NAFLD. Herein, we systematically evaluated the evidence regarding the efficacy of currently available GLP‐1 RAs on hepatic steatosis and fibrosis. Notably, the efficacy of GLP‐1 RAs on NASH was also evaluated.

#### 3.3.1 GLP‐1 RAs for the treatment of elevated hepatic enzymes

Patients with NAFLD with elevated hepatic enzymes are at higher risk of developing NASH, cirrhosis and end‐stage liver disease than those with normal enzymes.^50^ Importantly, sustained improvement in alanine aminotransferase (ALT) and aspartate aminotransferase (AST), together with improvement in hepatic steatosis, is a hallmark of reduced risk of progression to cirrhosis among NAFLD patients.^24^, ^25^, ^50^, ^51^, ^52^, ^53^ Therefore, the improvement in liver enzymes is the most commonly observed index in the study investigating the efficacy of GLP‐1 in the treatment of NAFLD.

Of 21 clinical trials reporting the change in hepatic enzymes as their end‐point, 19 studies supported the efficacy of GLP‐1 RAs on the improvement in hepatic enzymes (ALT, AST and GGT).^54^, ^55^, ^56^, ^57^, ^58^, ^59^ Feng et al conducted an RCT study involving a total of 87 patients and comparing the effects of liraglutide (n = 29), gliclazide (n = 29) and metformin (n = 29) for 24 weeks on body composition in patients with T2DM and NAFLD.^60^ In this study, both ALT and AST were markedly reduced in all three groups. However, there was no significant difference between groups. Liraglutide was also associated with a significant reduction in TG (2.73 ± 0.25 vs 1.83 ± 0.18 mmol/L, *P* < .01) and CHOL (4.86 ± 0.18 vs. 4.35 ± 0.15 mmol/L, *P* < .05). Consistent results were observed in an RCT study by Fan et al who investigated the effect of exenatide on blood glucose and hepatic enzymes in 117 patients with T2DM and NAFLD, suggesting that 12‐week treatment with exenatide was associated with a significant improvement in hepatic enzymes.^57^ In line with these findings, a retrospective study totalling 1499 participants evaluated the effects of dulaglutide (n = 971) versus placebo (n = 528) for 6 months on hepatic enzymes, indicating that ALT at the end of therapy in both groups was significantly reduced, with a greater reduction in the dulaglutide group.^53^ Collectively, these findings provide evidence for the efficacy of GLP‐1 RAs on the improvement in liver enzymes. Two studies, however, reported no relationship between GLP‐1 RA therapy and the change in hepatic enzymes.^61^, ^62^, ^63^ It should be noted that hepatic enzymes are not ideal markers of inflammation or damage to liver cells, as well as for the diagnosis and assessment of NASH, and the changes in hepatic enzymes are not necessarily parallel to liver histological alterations.^64^ Therefore, liver histological assessment is still needed when designing clinical trials to evaluate the efficacy of GLP‐1 RAs in the therapy of NASH.

#### 3.3.2 GLP‐1 RAs for the treatment of hepatic steatosis

GLP‐1 RAs have shown promise as a potential therapeutic option for improving hepatic steatosis in NAFLD.^63^, ^65^, ^66^, ^67^, ^68^ Improvement in hepatic steatosis determined by magnetic resonance spectroscopy (MRS) or magnetic resonance imaging proton density fat fraction (MRI‐PDFF) is one of the most critical primary end‐points for treatment trials designed to evaluate the efficacy of GLP‐RAs for NAFLD.^24^, ^25^, ^30^, ^31^, ^69^

Of 8 clinical trials reporting the change in hepatic steatosis as their end‐point, 6 studies demonstrated a significant reduction in liver fat content with GLP‐1 RA therapy. Cuthbertson et al, in a prospective study including 25 patients with a baseline therapy of metformin and sulphonylureas/dipeptidyl peptidase‐4, evaluated the effect of 6‐month GLP‐1 RAs (exenatide, n = 19; liraglutide, n = 6) on the intrahepatic lipid (IHL) measured by ^1^H MRS.^65^ In this study, GLP‐1 RA treatment was associated with a 42% relative reduction in IHL (−59.3, −16.5%) (*P* < .01), and the most considerable IHL reduction occurred among patients with highest pretreatment levels. Likewise, Dutour et al, in a prospective randomized trial enrolling a total of 44 obese subjects with T2DM randomly assigned to receive exenatide or reference treatment, found a substantial reduction in liver fat content in the exenatide group (−23.8 ± 9.5%) versus the reference group (+12.5 ± 9.6%) (*P* = .007).^63^ Participants in the exenatide group also had a more significant reduction in insulin resistance, as assessed by HOMA‐IR, and in total cholesterol compared with those in the reference group. Consistent with this study, Petit et al conducted a parallel study evaluating the effect of 6‐month treatment with liraglutide 1.2 mg/d on liver fat content in patients with uncontrolled T2DM. They found a mean reduction of 31% in liver fat content by ^1^H MRS (from 17.3 ± 10.9% to 11.9 ± 9.3%, *P* < .001), while no significant alteration of liver fat content occurred in the parallel group of patients who received intensification of the antidiabetic treatment with insulin.^66^ Aligned with these findings, Khoo et al conducted an RCT study involving 24 obese adults with NAFLD who were randomized to a group of dieting plus moderate‐intensity aerobic exercise (n = 12) or liraglutide at the 3 mg daily dose (n = 12) for 26 weeks. Both diet plus aerobic exercise and liraglutide significantly reduced the liver fat fraction (−8.9 ± 13.4%, *P* = .03; −7.2 ± 7.1%, *P* = .008, respectively), although there was no significant difference between two groups.^67^ Significant correlations were found between reduction from baseline in liver fat fraction with weight, waist circumference, fat mass and ALT. The reduction in HOMA was also linked to a reduction in weight, ALT and liver fat fraction. These studies, despite the small sample size, demonstrate the efficacy of GLP‐1 RAs on the improvement in hepatic steatosis.

The combined therapy of GLP‐1 RAs with oral antihyperglycaemic medications (OAMs) or insulin has been increasingly accepted in the treatment of T2DM because this combination not only improves glycaemic control but also avoids weight gain and an increased risk of hypoglycaemia.^70^ Moreover, several studies have been carried out to determine whether combined therapy can provide additional benefits than the single use of GLP‐1 RAs for hepatic steatosis. Sathyanarayana et al conducted an RCT study examining the effect of combined exenatide and pioglitazone therapy on liver fat content in patients with T2DM with diet or metformin as baseline treatment. 21 patients received either pioglitazone (45 mg/d, n = 10) or combined therapy with pioglitazone and exenatide (n = 11) for 12 months. Liver fat content was significantly reduced with pioglitazone treatment (11.0 ± 3.1 to 6.5 ± 1.9%, *P* < .05), and combined pioglitazone and exenatide therapy was linked to a more significant decrease in hepatic fat (12.1 ± 1.7 to 4.7 ± 1.3%, *P* < .05). Both groups significantly reduced the level of TG (136 ± 13 to 85 ± 7 mg/dL, *P* < .05 in the Exe plus Pio group; 192 ± 25 to 165 ± 19 mg/dL, *P* < .01 in Pio group), with a greater reduction in Exe plus Pio group (*P* < .01). Both treatments significantly decreased the level of hepatic inflammatory biomarkers (ALT and AST), with combined pioglitazone and exenatide therapy being associated with a more significant reduction in ALT.^71^ Consistent with the findings, Shao et al conducted an RCT study where 60 newly diagnosed patients with T2DM and NAFLD were randomly assigned into the exenatide group (exenatide and insulin glargine, n = 30) and the intensive insulin group (insulin aspart and insulin glargine, n = 30) for 12 weeks. They found the reversal rate of fatty liver determined by ultrasonography was significantly higher in the exenatide group than in the intensive insulin group (93.3% vs 66.7%, *P* < .01), as well as a significantly lower level of ALT, AST and GGT in the exenatide group than in the intensive insulin group (*P* < .001).^72^ Additionally, Blaslov et al conducted a 6‐month open‐label parallel‐group uncontrolled study using the fatty liver index (FLI) for noninvasively evaluating the liver fat content. They compared the effect of exenatide alone or in combination with oral hypoglycaemic agents (OHA) with OHA on liver fat content. The exenatide treatment was associated with a more significant change in FLI than in the OHA group, and the addition of exenatide to OHA therapy leads to a reduction in FLI.^62^

Totally, these findings provide evidence indicating that combination therapy of GLP1 RAs with other antidiabetic medicine not only offers the advantages of complementary pharmacologies with better glycaemic control but also leads to a greater improvement in hepatic steatosis than the single use of GLP‐1 RAs.

#### 3.3.3 GLP‐1 RAs for the treatment of hepatic fibrosis

The primary objective of treatment for NASH is to prevent the development of cirrhosis, and increasing hepatic fibrosis is the hallmark of disease progression to cirrhosis.^50^ Therefore, it is important to determine the effect of GLP‐1 RAs on the improvement in fibrosis when evaluating their role in the treatment of NAFLD.^24^, ^25^, ^50^ Most clinical trials examining the effect of GLP‐1 RAs on hepatic fibrosis used liver stiffness measured by transient elastography (TE) or magnetic resonance elastography (MRE) to assess the magnitude of fibrosis because liver stiffness has been validated as a reliable method for the assessment of liver fibrosis.^73^, ^74^, ^75^, ^76^, ^77^

A total of 4 clinical trials reported improvements in the noninvasive assessment of liver fibrosis as their end‐point. The method for noninvasively assessing the severity of fibrosis included serum markers (n = 2), TE (n = 1) and MRE (n = 1). Of 4 studies, three studies showed significant improvement in the magnitude of liver fibrosis with GLP‐1 RA therapy. In the study by Khoo et al who reported on the effect of dieting plus moderate‐intensity aerobic exercise (n = 12) or liraglutide (n = 12) on the liver fat fraction, they also examined the change in liver stiffness after 26‐week treatment and found that both groups had a significant reduction in liver stiffness (−0.21 ± 0.19, *P* = .001; −0.26 ± 0.29, *P* = .003, respectively), although there was no significant difference between groups. Likewise, Ohki et al, in a retrospective study enrolling 82 Japanese NAFLD patients with T2DM, compared the effect of liraglutide with sitagliptin and pioglitazone, suggesting a significant reduction in APRI score in the liraglutide and pioglitazone group, while there were no significant changes in the sitagliptin group.^78^ Again, a study by Seko et al who evaluated the effect of dulaglutide in 15 Japanese patients with biopsy‐proven NAFLD showed a similar result. In this study, 5 patients undergoing transient elastography at baseline and at the end of therapy with 12‐week dulaglutide treatment showed a significantly decreased liver stiffness (9.3 ± 1.9 to 6.9 ± 1.2 KPa, *P* = .043).^51^ The two remaining studies used the APRI score or FIB‐4 score to evaluate the severity of liver fibrosis. Ohki et al claimed a significant reduction in APRI score with liraglutide therapy in retrospective cohort studies, while an RCT by Smits et al failed to find any positive association of liraglutide or sitagliptin therapy with a reduction in APRI or FIB‐4 score.

Totally, despite the significant improvement in noninvasive assessment of liver fibrosis with GLP‐1 RA treatment, their role of GLP‐1 RAs for fibrosis regression and for preventing liver fibrosis from developing into liver cirrhosis remains unclear. Further, there is still a need for well‐designed prospective studies with long‐term follow‐up and improvement in biopsy‐proven fibrosis as the primary end‐point.

#### 3.3.4 GLP‐1 RAs as a potential therapeutic option for NASH

NASH is the most severe phase of NAFLD, characterized by the presence of an abnormal accumulation of fat, hepatocellular ballooning and inflammation, with or without fibrosis. Although many studies have shown the potential of GLP‐1 RAs for NAFLD, the evidence for the role of GLP‐1 RAs in the management of NASH remains inconclusive. Several studies have suggested no association existed between GLP‐1 RA treatment and the improvement in hepatic steatosis measured by noninvasive methods.^58^, ^79^, ^80^, ^81^ It should be noted that these studies used noninvasive methods rather than liver biopsy to diagnose and measure the changes in steatosis and fibrosis. Therefore, studies using liver biopsy to evaluate the histological changes in NASH patients with GLP‐1 RA treatment are still needed. Moreover, the resolution of steatohepatitis with no worsening of fibrosis is the most important end‐point for NASH treatment in clinical trials, with the highest level of evidence.^50^, ^82^ Again, NAFLD activity score (NAS), representing the sum of scores for steatosis (0‐3), lobular inflammation (0‐3) and hepatocellular ballooning (0‐2), has been established as a tool to measure histological changes in NAFLD during therapeutic trials.^83^ This further supported the importance of biopsy‐proven liver histology for evaluating the efficacy of GLP‐RAs on NASH in clinical trials.

Accumulating evidence suggests that GLP‐1 RAs show promise in the treatment of NAFLD, although there are few therapeutic studies with biopsy‐confirmed liver histological change as the primary end‐point. Four studies reported histological change with GLP‐1 RA therapy. A pilot study by Eguchi et al using a biopsy to evaluate the liver histology in 10 patients with T2DM and NAFLD found that 96‐week liraglutide therapy resulted in a biopsy‐proven histological inflammation improvement in 7 patients, while there was no difference in 2 patients and a worse Brunt classification grade in 1 patient. Meanwhile, improved liver fibrosis was found in 6 patients. Totally, 8 patients have improved NAS scores at 96 weeks compared with the first biopsy.^80^ However, this pilot study lacked a control group and thus cannot lead to a statistically significant conclusion, although liver biopsy was adopted in this single‐arm study. In a case series study including 8 adult patients with T2DM and biopsy‐proven NAFLD, there was no significant improvement in liver histopathology after 28 weeks of treatment with exenatide. However, these findings should be treated with caution because of the limited number of individuals included in this study and the lack of a control group.^84^ Given the limitation of single‐arm studies, the beneficial histological effect is needed to be examined in well‐controlled clinical trials. Armstrong et al conducted a multicentre, double‐blinded, randomized, placebo‐controlled phase 2 trial where 26 patients were assigned to receive liraglutide and 26 to placebo. After 48 weeks of treatment, 9 (39%) of 23 patients in the liraglutide group versus 2 (9%) of 22 participants in the placebo group had resolution of definite nonalcoholic steatohepatitis (relative risk = 4.3 [95%CI: 1.0‐17.7], *P* = .019). 2 (9%) of 23 patients who received liraglutide had progression of fibrosis compared with 8 (36%) of 22 patients in the placebo group (relative risk = 0.2 [0.1‐1.0], *P* = .04).^61^

Collectively, significant improvement in biopsy‐confirmed liver histology with GLP‐1 RA treatment provides the most substantial evidence for the efficacy of GLP‐1 RAs in the management of NASH, although the role of GLP‐1 RAs is still needed to be validated in large sample controlled trials with long‐term follow‐up.

## 4 ONGOING CLINICAL TRIALS

Database search identified 38 potentially relevant ongoing clinical trials: 30 were not related to fatty liver, and finally, 8 ongoing trials were included, involving 2 dulaglutide, 1 liraglutide and 5 semaglutide (Table 3). These 8 trials included 7 controlled trials (6 RCTs and 1 non‐RCTs) and 1 single‐arm trial. Four of the 8 trials plan to use liver histology as the primary end‐point, including NASH resolution without worsening of fibrosis and a reduction of at least 2 points in the NAFLD activity score. Three of 8 plan to use change in liver fat content on magnetic resonance imaging proton density fat fraction (MRI‐PDFF) or liver stiffness on MRE as the primary end‐point. One of 8 will use the number of treatment‐emergent adverse events, serious adverse events and any grade ≥ 1 laboratory abnormality as the primary end‐point. Of 7 controlled trials, 3 plan to use placebo, 2 use lifestyle intervention, and 2 use antidiabetic medicines or other medicine targeting NASH as their control. On review of ongoing trials, there has been a general shift from the use of hepatic enzymes and ultrasonographical findings as end‐points to MRI assessment of hepatic steatosis, liver fibrosis and biopsy‐confirmed liver histology.

**TABLE 3:** Undergoing clinical trials investigating the efficacy, safety and tolerability of GLP‐1 RAs in the treatment of NAFLD

	Title	Conditions	Interventions	Characteristics	Primary outcome	Secondary outcome	Location
NCT03590626	Effect of dulaglutide on liver fat in patients with type 2 diabetes and nonalcoholic fatty liver disease (D‐LIFT)	NAFLD T2DM	Dulaglutide Antidiabetic medicines	Randomized Phase: NA	Change in liver fat quantified by MRI‐PDFF	Changes in biochemical markers, LSM, CAP, etc	India
NCT03648554	Researching an effect of GLP‐1 agonist on liver steatosis (REALIST)	T2DM NASH	Dulaglutide Reinforced dietary monitoring	Randomized Phase 4	Regression of NASH without worsening of fibrosis	Changes in Kleiner score of fibrosis, fibrosis markers, and liver enzymes	France
NCT03987451	A research study on how semaglutide works in people with fatty liver disease and liver damage	NASH	Semaglutide Placebo	Randomized Phase 4	Relative change in liver stiffness measured by MRE	Relative change in LFC measured by MRI‐PDFF, NASH resolution, etc	United States
NCT03357380	A study on how semaglutide works on early stages of scar tissue in the liver assessed by pictures of the liver	Hepatobiliary disorders NAFLD	Semaglutide Placebo	Randomized Phase 1	Change in liver stiffness assessed by MRE	Change in liver stiffness assessed by MRE and in LFC by MRI‐PDFF, and proportion of subjects with at least 30% reduction in relative LFC.	Germany
NCT03884075	Nonalcoholic fatty liver disease, the hepatic response to oral glucose, and the effect of semaglutide (NAFLD HEROES)	NASH NAFLD	Semaglutide	Single‐group assignment Phase 2	Histological improvement（>=2 point decrease in NAFLD activity score） and clinical improvement	NA	United States
NCT02970942	Investigation of efficacy and safety of three dose levels of subcutaneous semaglutide once daily versus placebo in subjects with nonalcoholic steatohepatitis	Hepatobiliary disorders NASH	Semaglutide Placebo	Randomized Phase2	NASH resolution without worsening of fibrosis	liver fibrosis improvement, NAFLD activity score, etc	United States
NCT03987074	Safety, tolerability and efficacy of monotherapy and combination regimens in adults with nonalcoholic steatohepatitis (NASH)	NASH	Semaglutide Firsocostat Cilofexor	Randomized Phase 2	The number of treatment‐emergent adverse events and serious Adverse events (SAEs), and any grade ≥ 1 laboratory abnormality	NA	United States
NCT02654665	Comparing effects of liraglutide and bariatric surgery on weight loss, liver function, body composition, insulin resistance, endothelial function and biomarkers of nonalcoholic steatohepatitis (NASH) in obese Asian adults (CGH‐LiNASH)	NAFLD Weight Loss NASH	Liraglutide Lifestyle modification	Nonrandomized Phase 3	Improvement in NASH and reduction/normalization in transaminases and liver fat	NA	Singapore

Abbreviations: CAP, controlled attenuation parameter; LFC, liver fat content; LSM, liver stiffness measurement; MRE, magnetic resonance elastography; MRI‐PDFF, magnetic resonance imaging proton density fat fraction; NA, not applicable.

## 5 DISCUSSION

Early evidence of GLP‐1 RAs for NAFLD comes from studies reporting an improvement in hepatic enzymes with exenatide therapy.^59^, ^85^ These findings have been confirmed in many RCTs using GLP‐1 RAs to treat T2DM or NAFLD. Moreover, our conclusion on the effect of GLP‐1 RAs on elevated liver enzymes was similar to an individual patient data meta‐analysis of six 26‐week, phase‐III, randomized controlled TWD trials, which known as the ‘Liraglutide Effect and Action in Diabetes’ (LEAD) programme.^76^ Similarly, an individual patient data meta‐analysis of 15 RCT on patients with T2DM found that lixisenatide increased the proportion of obese or overweight patients who achieved normalization of ALT.^77^ Efficacy and safety of GLP‐1 RAs were also evaluated in a meta‐analysis indicating that GLP‐1 RAs may reduce aminotransferase levels and improve liver histology.^86^

This systematic review of clinical trials investigated the role of GLP‐1 RAs in the management of NAFLD. A total of 24 clinical trials, consisting of RCTs (n = 14, 58%) and other types of studies (n = 10, 42%), were included in this review. Of the 24 clinical trials identified consisting of 6313 individuals, there was significant heterogeneity in study design quality, sample size, duration, placebo choice and outcome measures. Data from clinical trials provide evidence that GLP‐1 RAs are effective in improving hepatic steatosis and inflammation. However, the potential of GLP‐1 RAs to regress fibrosis, as well as to prevent the progression of steatosis to NASH and cirrhosis, is still needed to be confirmed by prospective RCTs with more sensitive end‐points of hepatic fibrosis.

Multiple mechanisms are responsible for the development of NAFLD.^87^ Accumulation of fat from increased free fatty acid (FFA) uptake and de novo lipogenesis is an essential driving force for hepatic steatosis. On the other hand, decreased lipid removal from impaired fatty acid oxidation and VLDL secretion is also critically involved in the development of NAFLD. An unhealthy lifestyle, such as excessive caloric intake and the lack of exercise, leads to an increased level of FFA to the liver and an upregulated de novo lipogenesis (DNL). Moreover, insulin resistance in obese individuals results in unrestricted adipose tissue lipolysis, contributing to the flux of FFA from adipose tissue to the liver.^35^, ^36^ Adipose tissue with insulin resistance is one of the primary sources of pro‐inflammatory cytokines, including TNF‐α, IL‐1β and IL‐6, which play a vital role in the development of hepatic insulin resistance and NASH.^37^, ^38^, ^39^, ^40^ Circulating hormones secreted from adipose tissue, such as adiponectin, have also been implicated in the modulation of insulin resistance.^41^ Adiponectin is associated positively with insulin sensitivity, promoting fatty acid β‐oxidation (FAO), glucose use and suppression of fatty acid synthesis.^42^, ^43^ Patients with NAFLD have a lower level of adiponectin compared with BMI‐matched controls.^44^ Collectively, NAFLD is closely associated with both hepatic and adipose tissue insulin resistance, and reduced systemic insulin resistance (Figure 2).^45^, ^46^, ^47^, ^48^

GLP‐1 is secreted into the hepatic portal system by the intestinal L cells located primarily in the distal ileum and colon, stimulating insulin secretion in a glucose‐dependent fashion. GLP‐1 reduces glucagon output, delays gastric emptying and suppresses appetite, leading to a significant weight loss. Preclinical studies have demonstrated the efficacy of GLP‐1 agonists for the improvement in hepatic insulin sensitivity, steatosis and histology.^88^, ^89^, ^90^, ^91^ GLP‐1 improves insulin signal transduction in adipocytes by upregulating Akt phosphorylation and protein expression of cyclins A, D1 and E.^92^ Moreover, the direct effect of GLP‐1 RAs on hepatocytes has been validated by in vitro study where exenatide activated genes involved in hepatic fatty acid oxidation and insulin sensitivity in hepatocytes isolated from rats with NASH,^93^ although conflicting data still exist in terms of the presence of GLP‐1 receptors on human hepatocytes.^94^

This review has highlighted the limitations of the current data for the treatment of NAFLD. A major issue that hinders drug development for NAFLD is the need for biopsy‐confirmed liver histology to evaluate the severity of disease and assess response to therapies. It is critical to measure disease severity, particularly the presence of NASH and the stage of fibrosis because NASH and fibrosis severity have been strongly implicated in the long‐term prognosis of NAFLD. Although liver biopsy, in combination with Kleiner's histological NAFLD activity score (NAS), is still the gold standard for the stage of NASH, it is impractical to perform a liver biopsy in a large sample group because of the potential risk of infection and bleeding. Another limitation of liver biopsy is that the volume of a needle biopsy sample represents only a very minor fraction (1/50 000) of the entire liver, which can result in false negatives due to the heterogeneity of liver injury in NAFLD.

Of 24 clinical trials included in this systematic review, only 4 trials used liver histological change as their outcome measure. Due to the limitations of liver biopsy, complex molecular mechanisms underlying NASH and the long duration to progress into the advanced stage of the disease, it is challenging but necessary to develop meaningful surrogate end‐points.^95^, ^96^, ^97^, ^98^ Noninvasive modalities for assessment of NAFLD have been developed and increasingly used in clinical trials to define the end‐points. Magnetic resonance elastography (MRS) and MRI‐PDFF are emerging as useful imaging markers to assess treatment response in clinical trials in NASH.^30^, ^99^ Moreover, our conclusion is based on trials using not only the change in hepatic enzymes as the end‐point but also hepatic steatosis, inflammation and fibrosis as outcome measures, providing more comprehensive evidence of the efficacy of GLP‐1 RAs in the treatment of NAFLD than previous studies.

## 6 CONCLUSION

In this systematic review of published and ongoing clinical trials of the efficacy of GLP‐1RAs for NAFLD, we found that GLP‐1 RAs are effective for the improvement in hepatic enzymes and hepatic steatosis, with the potential to reverse fibrosis. More importantly, GLP‐1 RAs show promise in improving histological features of NASH, although the number of studies assessing the histological response to GLP‐1 RA therapy is limited. Further prospective studies of sufficient duration using histological end‐points are needed to fully assess the efficacy of GLP‐1 RAs in the management of NAFLD.

## CONFLICT OF INTEREST

The authors have declared no conflict of interest.

## AUTHOR CONTRIBUTIONS

XD lv, LL Hu and SY Qin designed the study. SY Qin wrote the manuscript. XD lv, YQ Dong, LL Hu and FY Lu searched databases, performed the selection of studies and assessed the quality of included studies. CY Zhou provided funding, designed the illustration and approved the last version.

## Supporting information

Supplementary Material 1

Supplementary Material 2

Supplementary Material 3

## Data Availability

All data related to this study are included in the manuscript and supplementary materials.
