# Supplementary material for: Glucagon‐like peptide‐1 receptor agonists (GLP‐1 RAs) for the management of nonalcoholic fatty liver disease (NAFLD): A systematic review
Source: Endocrinol Diabetes Metab. 2020 Jun 11;3(3):e00163. doi: 10.1002/edm2.163 (PMC7375121; doi:10.1002/edm2.163)
Supplement: Supplementary file 1 — Supplementary Material 1 [file EDM2-3-e00163-s001.docx]

**Supplementary material 01**

**Search strategy**

**PubMed**

#1 Glucagon-Like Peptide 1[MeSH Terms] 8,160

#2 ((((((Glucagon-Like Peptide 1[Title/Abstract]) OR dulaglutide[Title/Abstract]) OR exenatide[Title/Abstract]) OR liraglutide[Title/Abstract]) OR lixigenatide[Title/Abstract]) OR semaglutide[Title/Abstract]) OR albiglutide[Title/Abstract] 12,563

#3 (#1) OR #2 14,422

#4 Non-alcoholic Fatty Liver Disease[MeSH Terms] 10,682

#5 ((((Non-alcoholic Fatty Liver Disease[Title/Abstract]) OR Nonalcoholic Fatty Liver Disease[Title/Abstract]) OR steatosis[Title/Abstract]) OR Steatohepatitis[Title/Abstract]) OR fatty liver[Title/Abstract] OR hepatic fat[Title/Abstract] OR liver fat[Title/Abstract] 42,846

#6 (#4) OR #5 43,626

#7 (#3) AND #6 402

**Web of Science**

Database= WOS, KJD, MEDLINE, RSCI, SCIELO

# 1 TS=Glucagon-Like Peptide 1 22,102

# 2 TI= (Glucagon-Like Peptide 1 OR dulaglutide OR exenatide OR liraglutide OR lixigenatide OR semaglutide OR albiglutide) 9,213

# 3 #2 OR #1 25,109

# 4 TS=Non-alcoholic Fatty Liver Disease 26,443

# 5 TI= (Non-alcoholic Fatty Liver Disease OR Nonalcoholic Fatty Liver Disease OR steatosis OR Steatohepatitis OR fatty liver OR hepatic fat OR liver fat) 43,264

# 6 #5 OR #4 52,267

# 7 #6 AND #3 475

**Scopus**

#1 TITLE-ABS-KEY ( "Glucagon-Like Peptide 1" OR dulaglutide OR exenatide OR liraglutide OR lixigenatide OR semaglutide OR albiglutide ) 23,577

#2 TITLE-ABS-KEY ( "Non-alcoholic Fatty Liver Disease" OR "Nonalcoholic Fatty Liver Disease" OR steatosis OR steatohepatitis OR fatty AND liver OR "hepatic fat" OR "liver fat" )

#3 ( TITLE-ABS-KEY ( "Glucagon-Like Peptide 1" OR dulaglutide OR exenatide OR liraglutide OR lixigenatide OR semaglutide OR albiglutide ) ) AND ( TITLE-ABS-KEY ( "Non-alcoholic Fatty Liver Disease" OR "Nonalcoholic Fatty Liver Disease" OR steatosis OR steatohepatitis OR fatty AND liver OR "hepatic fat" OR "liver fat" ) )
