# Supplementary material for: Glucagon‐like peptide‐1 receptor agonists (GLP‐1 RAs) for the management of nonalcoholic fatty liver disease (NAFLD): A systematic review
Source: Endocrinol Diabetes Metab. 2020 Jun 11;3(3):e00163. doi: 10.1002/edm2.163 (PMC7375121; doi:10.1002/edm2.163)
Supplement: Supplementary file 2 — Supplementary Material 2 [file EDM2-3-e00163-s002.doc]

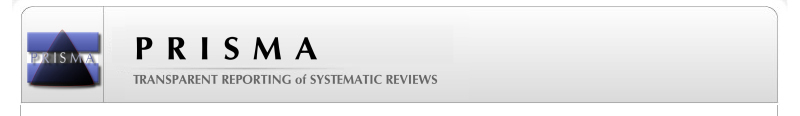
**PRISMA 2009 Flow Diagram**

**Screening**

**Included**

**Eligibility**

**Identification**

Records identified through database searching: PubMed, Web of Science and Scopus (n=1,933)

Records after duplicates removed
(n =1,461)

Potentially relevant articles
(n =109)

Records excluded after reading the abstracts (n=1,352)
(n =98)

Full-text articles assessed for eligibility

(n=24)

85 excluded on full-text for reasons:

⦁ Lack of relevance

⦁ Non-clinical trials

24 clinical trials included

⦁ Randomized controlled studies (n=14)

⦁ Paralleled-group uncontrolled studes (n=2)

⦁ Observational studies (n=1)

⦁ Retrospective studies (n=1)

⦁ Single-arm studies (n=3)

⦁ Case series studies (n=1)

⦁ Post-hoc analysis (n=2)
