# Supplementary material for: Glucagon‐like peptide‐1 receptor agonists (GLP‐1 RAs) for the management of nonalcoholic fatty liver disease (NAFLD): A systematic review
Source: Endocrinol Diabetes Metab. 2020 Jun 11;3(3):e00163. doi: 10.1002/edm2.163 (PMC7375121; doi:10.1002/edm2.163)
Supplement: Supplementary file 3 — Supplementary Material 3 [file EDM2-3-e00163-s003.docx]

**Supplementary material 3** Quality assessment of the randomized controlled studies based on the Cochrane Risk of Bias Tool

| Studies | Random sequence generation | Allocation concealment | Blinding of participants and personnel | Blinding of outcome assessment | Incomplete outcome data | Selective reporting | Other sources of bias |
| --- | --- | --- | --- | --- | --- | --- | --- |
| John,et al,2007 | **–** | **?** | **–** | **?** | **–** | **–** | **–** |
| Klonoff,et al,2008 | **–** | **?** | **–** | **?** | **–** | **–** | **–** |
| Jendle,et al ,2009 | – | ? | – | ? | – | – | – |
| Sathyanaryann,et al,2011 ealaal,20112015b[24] | – | ? | ? | – | – | – | – |
| Fan H,et al,2013 | – | ? | ? | ? | – | – | – |
| Shao,et al,2014 | – | ? | ? | – | – | – | – |
| Yan Bi, et al ,2014 | – | ? | ? | – | – | – | – |
| Tang, et al ,2015 | – | – | – | – | – | – | – |
| Armstrong, et al ,2016 | *–* | *–* | – | – | – | – | – |
| Smits, et al ,2016 | – | ? | – | – | – | – | – |
| Dutour, et al ,2016 | – | ? | – | – | – | – | – |
| Khoo, et al ,2017 | *–* | ? | + | – | – | – | – |
| Feng, et al ,2017 | *–* | ? | *–* | – | – | – | – |
| Tian.F, et al ,2018 | *–* | ? | ? | – | – | – | – |

+: High risk of bias.?: Unclear. −: Low risk of bias
